# Organic soil fertility management practices for the management of fall armyworm, *Spodoptera frugiperda* (J.E. Smith), in maize

**DOI:** 10.3389/finsc.2023.1210719

**Published:** 2023-09-26

**Authors:** Wakuma Bayissa, Asnake Abera, Jibril Temesgen, Gemechu Abera, Esayas Mendesil

**Affiliations:** Department of Horticulture and Plant Science, College of Agriculture and Veterinary Medicine, Jimma University, Jimma, Ethiopia

**Keywords:** crop residue, fall armyworm, manure, maize, pest management

## Abstract

The fall armyworm (FAW), *Spodoptera frugiperda* (J.E. Smith) (Lepidoptera: Noctuidae), is a polyphagous pest native to the Americas. It attacks several crops but in particular causes significant damage to maize, which is a staple crop in Africa. Organic soil amendments have an impact on the physical, chemical, and biological properties of soil, which enhance plant resistance to or tolerance of insect pests and also promote a diverse population of natural enemies of the pest. However, the practices followed for the management of crop residue and animal manure affect their use as organic soil amendments. A field experiment was conducted to evaluate the effect of maize residue and cattle manure incorporation into soil on FAW in the Mana and Omo Nada districts of the Jimma zone, southwest Ethiopia, during the 2018/19 cropping season. Treatment involved three factors: five different levels of maize residue retention (0%, 25%, 50%, 75%, and 100%), different cattle manure storage systems (control, open, steel roof, and grass roof), and two different districts (Mana and Omo Nada). These variables were organized in a randomized complete block design and replicated three times. The infestation and damage ratings were collected from 30 days after planting at 20-day intervals. The results indicated that maize plots with retained crop residue had a significant reduction in FAW infestation compared with plots without maize residue (control) in both study districts. Furthermore, manure-fertilized plants had a lower percentage of FAW infestation when compared with maize plots without cattle manure in both study districts. The lowest severity of FAW infestation was recorded in a plot with 100% of residue incorporated and treated with cattle manure stored under a grass roof in the Mana district. Therefore, conventional tillage with 100% maize residue incorporation and the application of cattle manure stored under a grass roof showed the best result for reducing FAW infestation in maize. However, further studies are important to determine the effect of treatments over seasons and locations on FAW infestation and maize yields.

## 1 Introduction

The fall armyworm (FAW), or *Spodoptera frugiperda* (J.E. Smith) (Lepidoptera: Noctuidae), is a native pest in the Americas. Its invasion of other regions was first detected in West Africa (1), and its occurrence has since been reported in more than 80 countries in Africa, Asia, and Oceania (2–5). FAW is a polyphagous pest that attacks more than 350 plant species (6). However, in Africa this pest primarily attacks maize, causing significant crop losses (7–11).

Given that maize is the most important staple crop for millions of people in the sub-Saharan Africa (SSA) region (12), large yield losses in maize caused by the invasion of FAW have further compounded the risks to food security in SSA. The invasion of FAW in Africa alarmed the governments of various countries, who then implemented a massive insecticide spray program to control FAW in maize fields (7, 11, 13, 14). However, dependence on chemical insecticides poses risks to human and environmental health and results in the development of insecticide resistance (15, 16). This suggests the need for an alternative FAW management approach that is sustainable and affordable for smallholder farmers.

As is common in most SSA countries, Ethiopian maize farmers are predominantly smallholder farmers who depend on cultural methods for the control of insect pests (7, 14, 17). According to a review by Harrison et al. (18) of native FAW infestation in the Americas, there are various cultural and agroecological practices, such as sustainable soil fertility management practices, tillage techniques, and cropping systems, which can help to manage FAW infestation. However, for invasive FAW, little information is available on the effectiveness of cultural practices for infestation management.

Organic soil fertility management practices, such as the use of crop residue and animal manure, not only improve soil fertility but also reduce insect pest infestation and improve crop productivity through different mechanisms (19–21). Organic soil fertility management practices maintain plant health by ensuring proper plant nutritional balance, which in turn enhances plant resistance to insect pest infestation (20, 22). In addition, crop residue and animal manure create favorable conditions for below- and above-ground microorganisms and macroorganisms that are naturally antagonistic toward and can regulate insect pest populations (20, 21, 23). For example, maize residue is important for soil health and soil microbial activities that encourage the growing of future crops by providing nutrients acquired by the previous plant (18, 24, 25). Similarly, the application of cattle manure reduces FAW infestation in maize crops (26) and decreases the performance of FAW (23).

Similar to most areas in Ethiopia, the farming system in the study area is predominantly a mixed crop–livestock system in which smallholder farmers primarily use maize stover as animal feed and fuel (27–29) and cattle manure as fuel (29, 30). This practice hinders the potential role of crop residue and cattle manure in improving soil fertility in the area. Furthermore, most farmers in the area do not use appropriate manure management practices, which results in the loss of important nutrients, such as nitrogen, before the manure is applied to the soil (31). Manure storage method is one of the most important factors that can influence nutrient retention (31, 32), manure quality, and nutrient content (33). Different manure storage systems appear to influence manure decomposition rates and nutrient loss owing to differences in temperature and moisture conditions (33). The current study aimed to explore the use of varying amounts of incorporated crop residue and the application of cattle manure managed under different storage systems for the suppression of FAW in the maize field.

## 2 Materials and methods

### 2.1 Description of the study area

**Figure 1:**
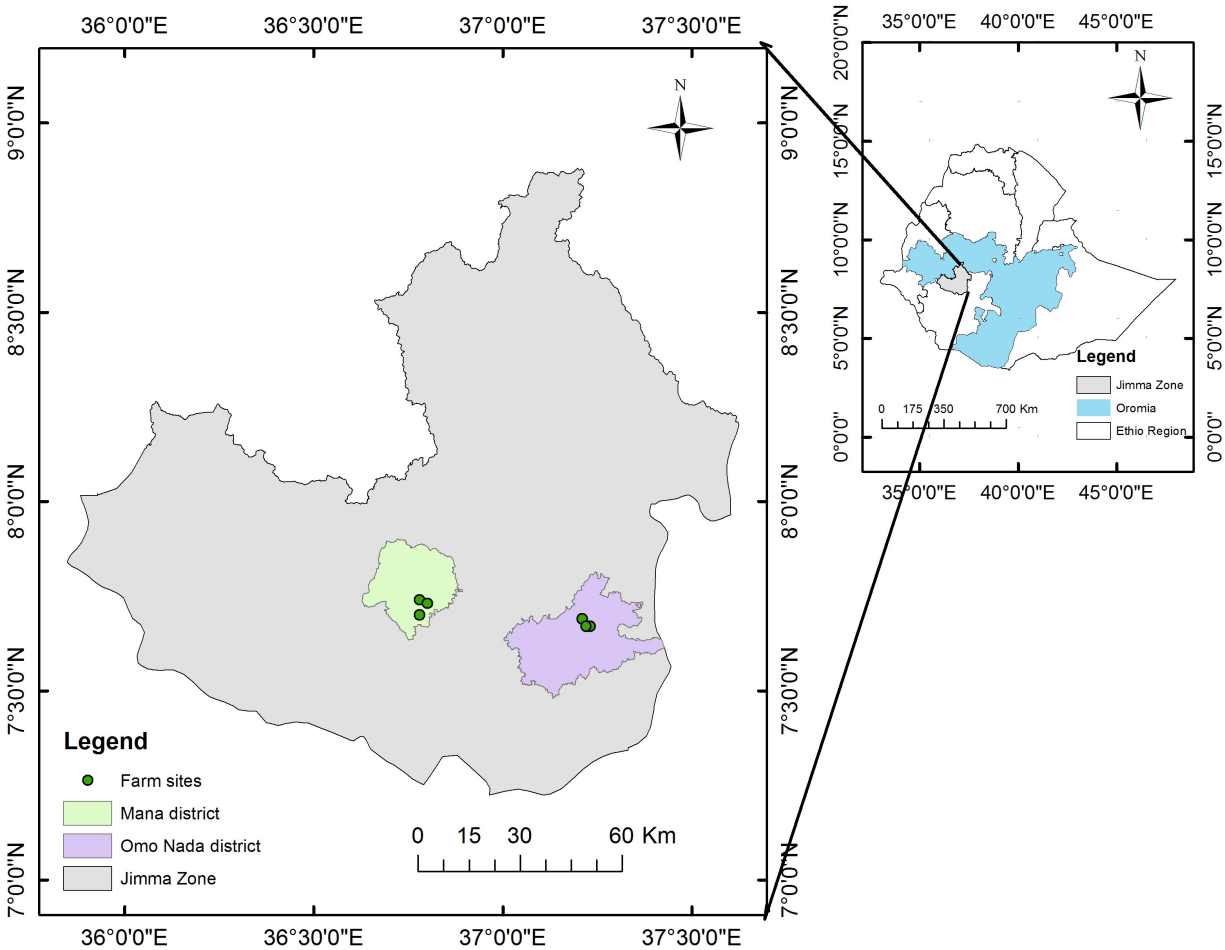
Map of the study sites in southwest Ethiopia.

The field experiment was carried out in the Mana and Omo Nada districts (**Figure 1**) of the Jimma Zone in southwest Ethiopia. The Omo Nada district is characterized by bimodal rainfall (unpredictable short rains from March to April) and the main rain season is between June and September. The mean annual rainfall ranges from 1,066 mm to 1,200 mm, and the mean annual temperature varies from 18°C to 25°C (34). The Mana district is characterized by a mean annual rainfall between 1,300 mm and 1,700 mm, with short spring rainfall (April and May) and long summer rainfall (June to August) seasons. The mean annual temperature ranges from 18°C to 20°C. The altitude, latitude, and longitude of the two areas area are summarized in **Table 1**.

**Table 1:** Description of the experimental sites.

Sites	Altitude (m.a.s.l.)	Latitude	Longitude
Omo Nada (Sayo Adami Peasant Association)			
Farm 1 (replication 1)	1,798	7°69′N	37°21′E
Farm 2 (replication 2)	1,774	7°69′N	37°21′E
Farm 3 (replication 3)	1,696	7°68′N	37°23′E
Mana (Somodo Peasant Association)			
Farm 1 (replication 1)	2,019	7°75′N	36°80′E
Farm 2 (replication 2)	1,922	7°74′N	36°78′E
Farm 3 (replication 3)	2,010	7°75′N	36°80′E

### 2.2 Experimental material

The maize variety BH661 used for the study was selected for its drought tolerance, high yield potential, and wide adaptability. It is a late-maturing maize variety released by the Bako Agricultural Research Center at the Ethiopian Institute of Agricultural Research in 2011. This variety is widely grown by smallholder farmers in the study area (35).

The cattle manure used in this study was obtained from the surrounding area and stored in steel- and grass-roofed houses with walls made of sticks that farmers had already constructed for animal housing. The maize residue used was obtained from previous maize crops.

### 2.3 Experimental design and treatment

Three different fields were used in each district in the middle of December 2018, which were chosen based on the accessibility of all manure storage systems (which were already constructed for animal housing purposes) and the availability of a fenced experimental plot that was previously occupied by the maize crop.

The total gross area of each field was 455.1m^2^, with a plot size of 15.36m^2^. The experimental fields were prepared using an oxen plow. The experimental fields were then tilled by inverting the topsoil using the oxen plow, thereby incorporating crop residue retained on the soil surface. The treatments were arranged in a randomized complete block design with three replicate fields and organized in a factorial arrangement. The experimental treatments included a factorial combination of five levels of maize residue incorporation (0%, 25%, 50%, 75%, and 100%) and four types of manure storage system (control without manure, open space, steel roof, and grass roof) in both the Mana and Omo Nada districts (**Table 2**). The experiment was replicated across three fields and the plots were randomly assigned to experimental units within a block. The maize was seeded manually with 80 cm spacing between rows and 40 cm spacing within rows. The number of rows per plot and the number of seeding holes per row were six and eight, respectively. Two maize seeds were seeded per hole at a depth of 5 cm.

**Table 2:** Treatment combinations of organic soil fertility management practices.

SN	Treatment
1	No residue × no animal manure
2	No residue × cattle manure stored in an open space
3	No residue × cattle manure stored under steel roof (SR)
4	No residue × cattle manure stored under grass roof (GR)
5	25% residue × no animal manure
6	25% residue × cattle manure stored in an open space
7	25% residue × cattle manure stored under steel roof (SR)
8	25% residue × cattle manure stored under grass roof (GR)
9	50% residue × no animal manure
10	50% residue × cattle manure stored in an open space
11	50% residue × cattle manure stored under steel roof (SR)
12	50% residue × cattle manure stored under grass roof (GR)
13	75% residue × no animal manure
14	75% residue × cattle manure stored in an open space
15	75% residue × cattle manure stored under steel roof (SR)
16	75% residue × cattle manure stored under grass roof (GR)
17	100% residue × no animal manure
18	100% residue × cattle manure stored in an open space
19	100% residue × cattle manure stored under steel roof (SR)
20	100% residue × cattle manure stored under grass roof (GR)

Treatments were replicated three times and organized in a factorial arrangement.

#### 2.3.1 Incorporation of residue in the soil

The maize residue used in this study was kept in the plots immediately after the previous maize crop was harvested and the site preparation was carried out. In plots with 96 previously sown maize plants, the following levels of residue incorporation were implemented: 0% (all residue removed), 25% (24 maize plants retained), 50% (48 maize plants retained), 75% (72 maize plants retained), and 100% (all 96 maize plants retained). The residue was chopped and incorporated at the relevant level during the first tillage. Any other biomass from above-ground weeds was completely removed from the experimental site and only maize residue was used for treatment. The crop residue was applied 3–4 weeks after the harvest of the previous maize crop, and the subsequent crop was sown approximately 18–19 weeks after incorporation of the residue. The experimental field was prepared using a local oxen plow before planting.

#### 2.3.2 Manure storage

Manure storage systems were determined from farmers’ practice in the study areas and considered as treatment levels (open, steel roof, and grass roof). The different manure treatments were used to capture the effects of the volatilization of nutrients due to solar heating (temperature). An impermeable plastic sheet (0.15-mm-thick polyethylene film) was used as lining underneath the manure (36).The cattle manure was stored for 5 months. Manure collection was completed in 3 days to eliminate time effects. Cattle manure was applied to the field at a concentration of 10 ton ha^−1^ (37) in both districts 2 weeks before the maize seeds were seeded and thoroughly incorporated into the soil in accordance with the assigned treatment.

#### 2.3.3 Crop management

The maize was seeded manually, with 80 cm spacing between rows and 40 cm spacing within rows. The number of rows per plot and the number of seeding holes per row were six and eight, respectively. Two maize seeds were seeded per hole at a depth of 5 cm. At the time of maize planting, blended fertilizer (NPSB) (18.1% N, 36.1% P_2_O_5_, 6.7% S, and 0.71% B) were applied to all plots at the recommended concentration (150 kg ha^−1^). The total maize planted was 62,500 plants ha^-1^ with respect to its plant spacing within the row spacing; and hoeing was carried out a week after emergence. Nitrogen fertilizer in the form of urea (46% N) was applied at a concentration of 200 kg ha^−1^ by split application: half at 40 days after planting and half before tasseling and immediately after weeding. After the crops reached full maturity, harvesting was carried out manually. All other necessary cultural practices, including hand weeding, were uniformly followed for all plots during the entire period of experimentation, when necessary.

### 2.4 Fall armyworm assessment

Foliar damage caused by FAW was recorded in 10 randomly sampled plants from each plot, taken from the four central rows. FAW observation was carried out until the maize reached physiological maturity 4 weeks after sowing. The number of maize plants infested with FAW was recorded at 35, 60, 90, and 120 days after sowing. The level of damage was rated on a 1–9 scale (38), where 1 was a plant with no visible damage and 9 was a completely damaged plant. The percentage of infested maize plants owing to FAW was calculated using the formula:


infestation percentage = (number of infested plants/total number of plants assessed) × 100

### 2.5 Data analysis

All data were checked for normality and homogeneity of variance prior to analysis. The data regarding FAW infestation and the insect damage ratings were not normally distributed. Therefore, data were analyzed by generalized linear mixed models (GLMMs) using a log link function with a gamma probability distribution to accommodate variations. Plots were included in the model as a random effect and the treatments as fixed effects. Mean comparisons between treatments were made using the least significant difference test. All analyses were performed using the SAS version 9.3 statistical software package (39).

## 3 Results

### 3.1 Fall armyworm infestation

**Table 3** shows the infestation of FAW in maize subjected to different soil management treatments. The percentage of FAW infestation was significantly affected by the study district, the level of maize residue, and the type of manure storage (*p* < 0.05). Moreover, the interactions between manure storage type and residue level, manure storage type and district, and residue level and district had an effect on FAW infestation. However, the interaction of study district, level of maize residue, and type of manure storage had no effect on FAW infestation. The smallest percentage of FAW infestation was recorded in Manna (15.4%) and Omo Nada (24.1%) in the plots treated with a maize residue level of 100%. This contrasts with the plots without maize residue, where 26.4% and 42.5% FAW infestation were recorded in the Mana and Omo Nada districts, respectively. However, there were no significant differences in FAW infestation between the plots with no maize residue and either those with a maize residue level of 25%–75% in Mana or those with a maize residue level of 25%–50% in Omo Nada.

**Table 3:** Infestation of fall armyworm in maize managed using different organic soil fertility management practices in southwest Ethiopia, 2019.

Residue management (%)	FAW infestation (%) ± SD	
	Mana	Omo Nada
0	26.4 ± 5.8^cd^	42.5 ± 5.5^a^
25	24.7 ± 8.4^cde^	37.5 ± 9.8^ab^
50	22.7 ± 10.8^cde^	33.1 ± 13.6^abc^
75	20.8 ± 13.0^de^	29.1 ± 17.6^bcd^
100	15.4 ± 16.1^e^	24.1 ± 20.7^cde^
LSD (0.05)	10.6	
CV %	10.2	
Manure storage system		
Am0	37.3 ± 4.5^b^	54.5 ± 4.1^a^
Am1	22.6 ± 5.6^de^	30.5 ± 10.4^c^
Am2	17.3 ± 8.0^e^	26.1 ± 9.4^cd^
Am3	10.8 ± 7.0^f^	22.0 ± 10.3^de^
LSD (0.05)	5.68	
CV %	10.21	

Am0, control (without cattle manure); Am1, cattle manure stored in open space; Am2, cattle manure stored under steel roof'; Am3, cattle manure stored under grass roof; CV, coefficient of variation; LSD, least significant difference. Mean ± standard deviation (SD) values within the same column followed by the same letters do not differ significantly at *p* = 0.05.

The smallest percentage of FAW infestation was observed in plots treated with cattle manure stored under a grass roof in both Mana (10.8%) and Omo Nada (22%), contrasting with plots without cattle manure in both Mana (37.3%) and Omo Nada (54.5%). Except for the cattle manure stored under the grass roof, there was no significant difference in FAW infestation between types of cattle manure storage (**Table 3**).

### 3.2 Severity of fall armyworm infestation

The severity of FAW infestation was significantly affected by the study district and maize residue level (*p*<0.001); the manure storage type (*p*<0.001); and the interaction effect of district, the level of maize residue, and the manure storage type (*p*<0.001) (**Table 4**). The severity of FAW infestation ranged from 0.01 to 1.25 and from 0.86 to 1.6 in the Mana and Omo Nada districts, respectively. The highest severity of FAW infestation (1.60) was recorded in the control plot in the Omo Nada district and the lowest (0.01) was recorded in a plot with 100% of residue incorporated and the applied cattle manure stored under a grass roof in the Mana district (**Table 4**).

**Table 4:** Severity of fall armyworm natural infestation in maize managed using different organic soil fertility management practices in southwest Ethiopia, 2019.

Residue management (%)	Manure storage system	FAW damage ± SD	
		Mana	Omo Nada
0	Am0	1.25 ± 0.09^de^	1.60 ± 0.22^a^
	Am1	1.05 ± 0.17^fgh^	1.28 ± 0.20^cde^
	Am2	0.85 ± 0.11^klm^	1.16 ± 0.11^ef^
	Am3	0.65 ± 0.06^op^	1.03 ± 0.12^fghij^
25	Am0	53.06 ± 2.99^f^	1.44 ± 0.09^b^
	Am1	0.75 ± 0.05^mno^	1.04 ± 0.04^fghi^
	Am2	0.68 ± 0.08^no^	1.04 ± 0.05^fghi^
	Am3	0.43 ± 0.12^qr^	1.10 ± 0.10^fg^
50	Am0	0.82 ± 0.05^lmn^	1.41 ± 0.08^bc^
	Am1	0.52 ± 0.00^pq^	0.99 ± 0.00^ghijk^
	Am2	0.45 ± 0.02^qr^	0.99 ± 0.00^ghijk^
	Am3	0.25 ± 0.05^st^	0.99 ± 0.00^ghijk^
75	Am0	0.76 ± 0.03^mno^	1.30 ± 0.18^bcde^
	Am1	0.45 ± 0.09^qr^	0.99 ± 0.00^ghijk^
	Am2	0.22 ± 0.06^st^	0.96 ± 0.02^ghijkl^
	Am3	0.15 ± 0.02^tu^	0.93 ± 0.05^hijkl^
100	Am0	0.70 ± 0.09^no^	1.33 ± 0.08^bcd^
	Am1	0.36 ± 0.12^rs^	0.93 ± 0.05^hijkl^
	Am2	0.11 ± 0.01^tu^	0.88 ± 0.00^jklm^
	Am3	0.01 ± 0.02^u^	0.86 ± 0.01^klm^
F	10.815		
DF	12		
P	0.001		

Am0, control (without cattle manure); Am1, cattle manure stored in open space; Am2, cattle manure stored under steel roof; Am3, cattle manure stored under grass roof; DF, degree of freedom. Mean± standard deviation (SD) values within the same column followed by the same letters do not differ significantly at *p* = 0.05.

**Figure 2:**
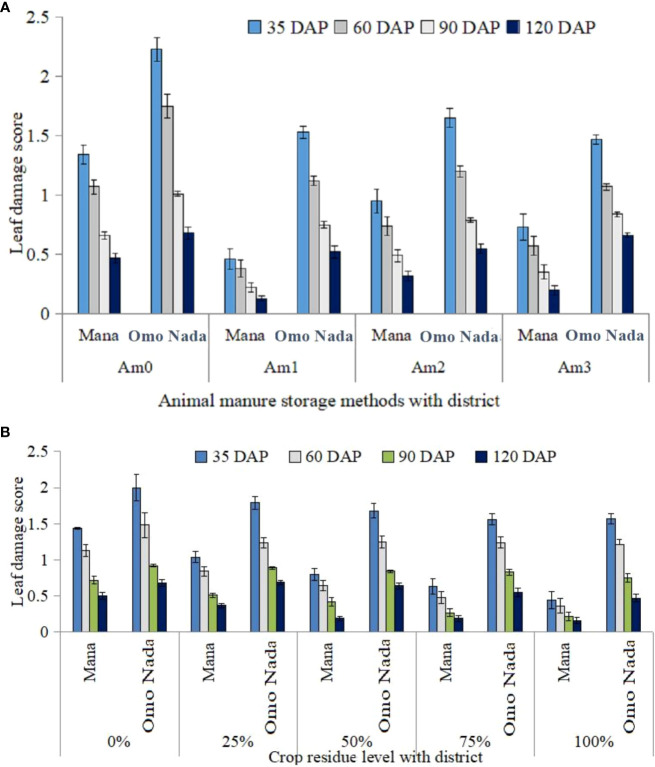
Leaf damage scores at different phonological stage of maize under natural infestation of FAW on maize managed in different organic soil fertility management practices **(A)** Animal manure **(B)** Maize residue in south west Ethiopia, 2019. Where: Am0= control (without cattle manure), Am1= cattle manure stored at open space, Am2= cattle manure stored under steel roof, Am3= cattle manure stored under grass roof, FAW=fall armyworm, DAP=days after planting, 0-100% maize residue incorporated into the soil.

**Figure 2** shows the severity of FAW infestation at different phenological stages for the maize. The severity of FAW infestation was significantly affected by the study district, the level of maize residue, and the type of manure storage (*p* < 0.05). Moreover, the interactions between manure and district and between residue and district had an effect on FAW severity. However, the interaction of the study district, the level of maize residue, and the type of manure storage had no effect on FAW severity. As shown in **Figure 2**, the highest severity of FAW infestation was observed in the early stages of maize growth and, after a certain period of time, the severity of FAW infestation declined and the maize quickly recovered from the damage. The highest leaf damage rating was recorded 35 days after planting (leaf stages 6–8), while few instances of leaf damage were recorded 90–120 days after planting or at the maturity stage. The severity of FAW infestation decreased as the level of residue incorporation increased, regardless of manure storage type (open, steel roof, and grass roof) or district (**Figure 2**; **Table 4**).

## 4 Discussion

The present study confirmed the low FAW infestation rates and damage caused by FAW in maize owing to organic soil fertility management practices under farmer field conditions. The retention of crop residue in maize plots significantly reduced FAW infestation in both study districts compared with plots without maize residue (control). The level of FAW infestation decreased with the increase of the proportion of maize straw retention as crop residue, as demonstrated by the 100% residue retention in the plots with the lowest percentages of FAW infestation in both the Manna and Omo Nada districts. The results also showed that the retention of maize residue and the application of cattle manure significantly reduced the severity of FAW infestation in maize plots in both districts compared with plots without residue retention and manure application.

Reduced levels of FAW infestation in plots with retention of crop residues may be attributed to the benefits obtained from crop residue in terms of improving soil fertility, which may have contributed to crop vigor or the resistance of maize plants to FAW damage. Crop residue retention is one of the agricultural conservation practices that improves the soil’s chemical, physical, and biological properties (22). As pointed out by Altieri and Nicholls, (20), organic soil fertility management practices are of paramount importance in maintaining plant health by ensuring proper plant nutritional balance and thereby increasing plant resistance to insect pests. Furthermore, according to the same authors, organic management practices create favorable conditions for pests’ natural enemies, which reduce insect pest infestation and minimize crop damage.

Although little information is available on the effect of crop residue incorporation on the invasive FAW species, including for Ethiopia, the retention of crop residues contributes to the suppression of insect pests, including FAW. For example, Rivers et al. (40) and recent reviews by Harrison et al. (18) demonstrated the role of crop residue retention in suppressing FAW infestation and improving the diversity and abundance of natural enemies of FAW in their native range. Studies have also shown that crop residue suppresses weeds and improves maize yields (e.g., 24, 25, 41–43). However, smallholder farmers often use crop residue for different purposes, such as livestock feed (44). In Ethiopia, maize stover is used mainly as a household energy source (fuel) and as livestock feed (27, 28). Such competing uses of crop residue are a major challenge for the use of crop residue as mulch in soil management (27, 28) and for the management of FAW (18).

The present study also showed that manure-fertilized plants exhibit a lower percentage of FAW infestation than plots without manure, with a significantly lower FAW infestation in plants fertilized with cattle manure stored under a grass roof in Mana (10.8%) and Omo Nada (22%). Grass roofs may have created a conducive environment for improved manure quality; this is in line with the findings of Tittonell et al. (33), who observed a positive effect from roofing on the quality and nutrient concentration of manure owing to the modification of the microclimate (i.e., the temperature and moisture) of the storage system. According to the same authors, roofing minimizes ammonia volatilization, which causes a considerable loss of N due to microclimate changes in the storage conditions that affect the decomposition rate of manure and nutrient loss. Maize plots treated with manure stored under a grass roof may have had better soil fertility, which may have contributed to reduced susceptibility to FAW damage, compared with plants treated with manure stored under a steel roof or in an open space. This result demonstrates the contribution of manure application to reducing FAW infestation under field conditions and corroborates the findings of Baudron et al. (26), who found lower FAW damage levels in maize plots with manure application, and Rowen and Tooker (23), who observed a decrease in the performance of FAW fed manure-fertilized corn.

Organic soil fertility management practices, such as the use of livestock manure, reduce insect pest infestation (18, 19, 23, 26, 45) through different mechanisms by enhancing plant resistance to insect pest infestation. Organically managed soil in general shows lower susceptibility to insect pests owing to proper mineral balance (46). In addition, by reviewing the results of several studies, Reddy (47) noted that the application of nitrogen fertilizers resulted in increased insect pest damage. Thus, sustainable soil fertility management practices are essential to improve soil fertility, improve plant health, and reduce susceptibility to insect pest attack (18, 21). Furthermore, manure improves the above-ground and below-ground interactions of microorganisms and macroorganisms that increase the presence of insect pests’ natural enemies, which in turn regulates the insect pest population (20, 21).

The results of this study demonstrate that maize residue retention and the application of cattle manure can reduce FAW damage, and this can be used for the cultural management of FAW by smallholder maize growers. However, long-term field trials will be important to understand the effects of crop residue retention and the application of manure on suppressing FAW infestation and on maize productivity.

## Data availability statement

The raw data supporting the conclusions of this article will be made available by the authors, without undue reservation.

## Ethics statement

Ethical review and approval was not required for the study on animals in accordance with the local legislation and institutional requirements.

## Author contributions

Conceptualization: WB, AA, GA, JT, and EM. Methodology: WB, AA, GA, JT, and EM. Software: GT and WB. Validation: JT and EM. Formal analysis: AA, GA, and EM. Data curation: AA, GA, and GT. Writing—original draft preparation: AA and GA. Reviewing: WB and EM. Project administration: JT. Funding acquisition: WB and GT. All authors contributed to the article and approved the submitted version.
